# Phytochemical Characterization and Evaluation of the Antimicrobial, Antiproliferative and Pro-Apoptotic Potential of *Ephedra alata* Decne. Hydroalcoholic Extract against the MCF-7 Breast Cancer Cell Line

**DOI:** 10.3390/molecules24010013

**Published:** 2018-12-20

**Authors:** Corina Danciu, Delia Muntean, Ersilia Alexa, Claudia Farcas, Camelia Oprean, Istvan Zupko, Andrea Bor, Daliana Minda, Maria Proks, Valentina Buda, Monica Hancianu, Oana Cioanca, Codruta Soica, Sofia Popescu, Cristina Adriana Dehelean

**Affiliations:** 1 Department of Pharmacognosy, University of Medicine and Pharmacy “Victor Babeş“, Eftimie Murgu Square, No. 2, 300041 Timişoara, România; corina.danciu@umft.ro; 2 Department of Microbiology, University of Medicine and Pharmacy “Victor Babeş“, Eftimie Murgu Square, No. 2, 300041 Timişoara, România; muntean.delia@umft.ro; 3 Department of Food Control, Banat’s University of Agricultural Sciences and Veterinary Medicine “King Michael I of Romania” from Timisoara, Calea Aradului No. 119, 300641 Timisoara, Romania; alexa.ersilia@yahoo.ro (E.A.); sofiapopescu@yahoo.com (S.P.); 4 Department of Pharmaceutical Physics, University of Medicine and Pharmacy “Victor Babeş“, Eftimie Murgu Square, No. 2, 300041 Timişoara, România; farcas.claudia@umft.ro; 5 Department of Drug analysis; chemistry of the environment and food, University of Medicine and Pharmacy “Victor Babeş“, Eftimie Murgu Square, No. 2, 300041 Timişoara, România; camelia.oprean@umft.ro; 6 OncoGen Centre, County Hospital "Pius Branzeu", Blvd. Liviu Rebreanu 156, 300736 Timisoara, Romania; 7 Department of Pharmacodynamics and Biopharmacy, University of Szeged, Eötvös u. 6., H-6720 Szeged, Hungary; zupko@pharm.u-szeged.hu (I.Z.); andrea.bor@pharm.u-szeged.hu (A.B.); 8 Department of Pharmacology and Clinical Pharmacy, University of Medicine and Pharmacy “Victor Babeş“, Eftimie Murgu Square, No. 2, 300041 Timişoara, România; proks.maria@yahoo.ro; 9 Department of Pharmacognosy, Faculty of Pharmacy, “Grigore T.Popa” University of Medicine and Pharmacy, 700115 Iasi, Romania; mhancianu@yahoo.com (M.H.); oana.cioanca@gmail.com (O.C.); 10 Department of Pharmaceutical chemistry, University of Medicine and Pharmacy “Victor Babeş“, Eftimie Murgu Square, No. 2, 300041 Timişoara, România; codrutasoica@umft.ro; 11 Department of Toxicology, University of Medicine and Pharmacy “Victor Babeş“, Eftimie Murgu Square, No. 2, 300041 Timişoara, România; cadehelean@umft.ro

**Keywords:** *Ephedra alata* Decne, polyphenols, antioxidant, bacteria, fungi, MCF-7 human breast cancer cell line

## Abstract

*Ephedra alata* Decne. belongs to the *Ephedraceae* family. It is a species of Ephedra that grows mostly in the desert. Today, the main importance of *Ephedra* species in the medical field is due to the presence of the alkaloids derived from phenyl-alanine, which act on the sympathetic nervous system as a sympathomimetic. The aim of this study was to conduct a phytochemical characterization of the hydroalcoholic extract of the aerial part of *Ephedra alata* Decne., which is indigenous to Tunis, that involves the total phenolic content, individual phenolic content, and antioxidant activity as well as a biological screening for the evaluation of the antimicrobial, antifungal, antiproliferative, pro-apoptotic, and cytotoxic potential against the MCF-7 breast cancer cell line. The results show that the hydroalcoholic extract contains polyphenolic phytocompounds (156.226 ± 0.5 mgGAE/g extract) and elicits antioxidant activity (7453.18 ± 2.5 μmol Trolox/g extract). The extract acted as a bacteriostatic agent against all tested bacterial strains, but was bactericidal only against the Gram-positive cocci and *Candida* spp. In the set experimental parameters, the extract presents antiproliferative, pro-apoptotic, and cytotoxic potential against the MCF-7 human breast cancer cell line.

## 1. Introduction

Throughout history, the Plant Kingdom has represented a significant source for the discovery of new drugs with important therapeutic effects in different areas of medicine. Various types of plant extracts include a large range of phytochemicals, which can be—on their own or through a synergistic mechanism—useful for different therapeutic activities. At the moment, many of the currently used drugs in well-established therapeutic protocols are directly obtained from, or are chemical derivatives of, phytochemicals [1].

*Ephedra alata* Decne. belongs to the *Ephedraceae* family. It is a species of Ephedra that grows mostly in the desert. The genus Ephedra is known to comprise approximately 40 species that populate arid environments, especially those from the northern hemisphere and the south part of America [2]. *Ephedra* species have a long history in traditional Chinese medicine (approximately 5000 years), with uses in the treatment of allergies, nasal congestion, bronchial asthma, coughs, and flu [3]. The drug is known under the name of Ma-huang. The drug was traditionally obtained from the dry stems of three *Ephedra* species, namely *Ephedra sinica, Ephedra intermedia*, and *Ephedra equisetina*, and is nowadays sold in health food stores in the West as a herbal mixture, known under the name of Herbal Ecstasy, that is proclaimed to have energizing value [4]. Today, the main importance of *Ephedra* species in the medical field is due to the presence of the alkaloids derived from phenyl-alanine (e.g., ephedrine and other related compounds, such as pseudoephedrine, norpseudoephedrine, norephedrine, methylephedrine, and methylpseudoephedrine), which acts on the sympathetic nervous system as a sympathomimetic [5]. It was reported in the literature that the aerial parts of this plant contain between 0.02 and 3.4% nitrate compounds, with (−)ephedrine being the main isomer detected (between 30 and 90%) [6]. Ephedrine is a medicine that is used to prevent arterial hypotension during spinal anesthesia. It is commonly employed as nasal decongestant, and has appetite-suppressant properties [7]. Besides this class of phytochemicals, the plant extract represents a source of polyphenolic compounds, which provide it with significant antioxidant properties [8]. Flavones, flavanols, bisflavanols, and carboxylic acids were reported to be the main phytochemicals along with alkaloids by the group of Abourashed et al. [6]. Also, essential oil has been indicated to be among the main components of this plant [9].

The literature describes some studies on the biological activity of *Ephedra* species. Extracts or fractions from callus cultures or wild plants from different species of *Ephedra* were reported to have antioxidant, antimicrobial, and antifungal properties [10,11]. Pullela et al. have isolated four lignans from *Ephedra viridis* Coville and screened these compounds for antioxidant and estrogenic activity and a possible cytotoxic effect against a number of human leukemia cells and solid tumors. The study concluded that the compounds elicited moderate free radical scavenging properties, no cytotoxicity, and estrogenic activity [12]. Nam et al. have shown that a water fraction of *Ephedra sinica* Stapf. has manifested anti-invasive, antiangiogenic, and antitumour effects in a mouse model of murine melanoma [13]. The hydroalcoholic extract obtained from *Ephedra campylopoda* C.A.Mey. has been assigned antioxidant, antibacterial, and in vitro antiproliferative properties against the HT 29 and HCT 116 colon cancer cell lines [14]. *Ephedra fragilis* Desf. extract elicited immunostimulant activity in an in vitro model using human peripheral lymphocytes [15]. *Ephedra alata* L. turned out to be a promising agent for a biologically based strategy for the inhibition of growth as well as aflatoxin production by *Aspergillus flavus* mold [16].

The aim of this study was to conduct a phytochemical characterization of the hydroalcoholic extract of the aerial part *Ephedra alata* Decne., which is indigenous to Tunis, that involves the total polyphenols content, individual polyphenols content, and antioxidant activity as well as a biological screening for an evaluation of the extract’s antimicrobial, antifungal, antiproliferative, pro-apoptotic, and cytotoxic potential against the MCF-7 breast cancer cell line.

## 2. Results

### 2.1. Phytochemical Composition

In order to determine the phytochemical composition, the total phenolic content (TP), individual phenolic content, antioxidant activity (AA), and total alkaloids (TA) were screened. The phytochemical composition of the hydroalcoholic extract of the aerial part of *Ephedra alata* Decne. (EA), determined by UV-VIS spectrometry, is presented in Table 1. The individual polyphenols and LC-MS parameters of the hydroalcoholic extract of the aerial part of *Ephedra alata* Decne. (EA) are presented in Table 2.

**Figure 1:**
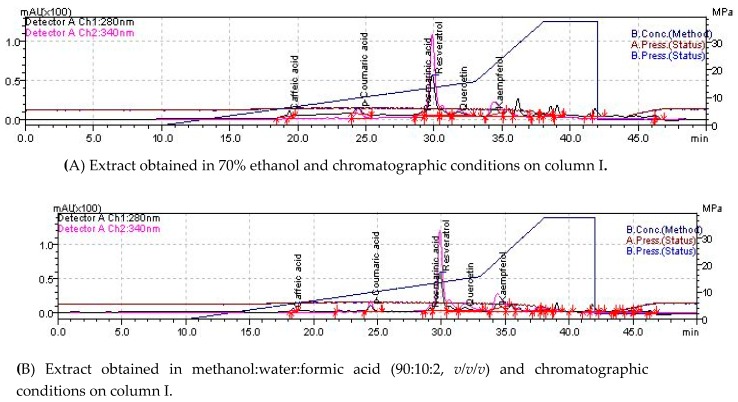
An LC-MS chromatogram of the hydroalcoholic extract of the aerial part of *Ephedra alata* Decne. (EA).

The individual polyphenols were detected using different extraction conditions and identified on two different chromatographic columns (Figure 1 and Table 2). The screened compounds were gallic acid, protocatecuic acid, caffeic acid, coumaric acid, ferulic acid, rosmarinic acid, epicatechin, rutin, resveratrol, quercetin, and kaempherol.

In the ethanolic extracts of EA, individual polyphenols were determined using two different C18 chromatographic columns under the same operating conditions. The compounds identified on both columns were: rosmarinic acid (mean value, 0.013 μg/mg), resveratrol (0.223 μg/mg), quercitin (2.63 µg/mg), and kampherol (15.55 µg/mg). Caffeic acid (0.014 µg/mg) and p-coumaric acid (0.05 µg/mg) were identified in small quantities. These compounds were identified only on the Adsorbosphere UHS C18 column, while epicatechin was identified on the NUCLEODUR C18 Gravity SB column.

In the methanolic extract, rosmarinic acid (0.016 µg/mg), resveratrol (0.207 µg/mg), quercitin (0.091 µg/mg), kampherol (2.867 µg/mg), caffeic acid (0.008 µg/mg), and p-coumaric acid (0.005 µg/mg) were detected. Gallic acid, protocatechuic acid, and ferulic acid were not detected in either of the two analyzed extracts.

### 2.2. Antimicrobial Activity

Another aim of the study was to screen EA for antimicrobial activity. The antibacterial activity of this extract was established according to the standardized value of the positive control (15 mm for gentamycin and 17 mm for fluconazole). All Gram-negative bacilli strains and *Enterococcus faecalis* were resistant to the test extract. The *Staphylococcus aureus* and *Candida* strains had insignificant inhibitory activity at the screened concentration. The inhibition diameters are presented in Table 3.

Table 4 presents the results on the MIC and MBC. The MIC values for S. aureus and Candida spp. (50 μg/mL) were lower than those for Gram-negative bacteria (200 μg/mL) and E. faecalis (100 μg/mL). In addition, the MBC values were 2 times greater than the MIC values. Based on these results, we can affirm that the extract can act as a bacteriostatic agent against all tested bacterial strains; however, it is bactericidal only against the Gram-positive cocci and Candida spp.

### 2.3. Anticancer Activity

**Figure 2:**
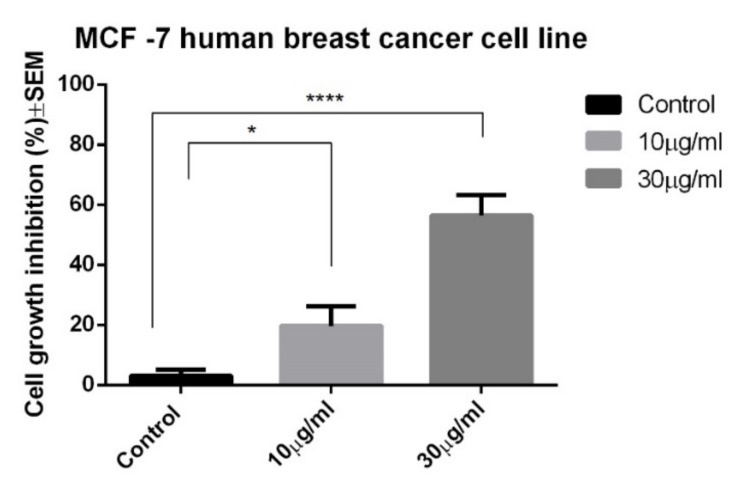
The cell growth inhibition for the MCF-7 human breast cancer cell line after 72 h of incubation with EA. SEM, standard error of the mean.

The EA was screened for possible in vitro anticancer activity against the MCF-7 human breast cancer cell line. The antiproliferative activity of EA at the selected concentrations after a period of incubation of 72 h is shown in Figure 2. Statistically significant results were detected starting from the concentration of 10 μg/mL, with a cell growth inhibition percentage of 19.68 ± 4.2. For the highest tested concentration, namely 30 μg/mL, the growth inhibition percentage was 56.45 ± 3.9.

**Figure 3:**
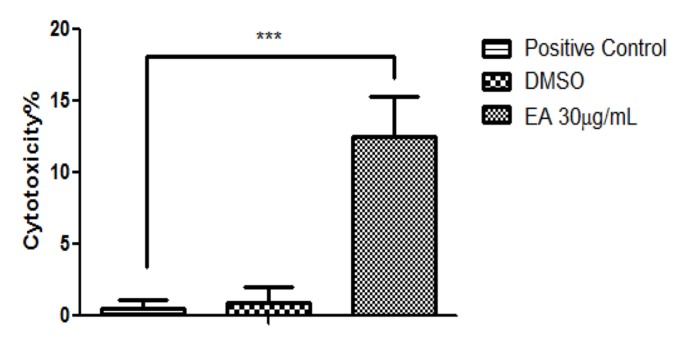
The cytotoxicity analysis of EA extract at a concentration of 30 μg/mL on MCF-7 cells (after 72 h of stimulation).

As is already well-known, MTT is a colorimetric assay for assessing the metabolic activity of cells (the activity of mitochondrial dehydrogenase). In order to see if we can also talk about a possible cytotoxic effect, the release of lactat dehydrogenase at the highest tested concentration was also measured. The results are depicted in Figure 3.

The cytotoxicity assessment revealed that the EA extract manifested a significant difference in cytotoxic potential when compared with the positive control, displaying a cytotoxicity percentage above 13%. The solvent (DMSO) showed no significant cytotoxic potential on MCF-7 cells.

The distribution of the phases of the cell cycle following incubation with EA at the concentration of 30 µg/ml shows a slight increase in the percentage of cells of the G0/G1 phase (data not shown).

**Figure 4:**
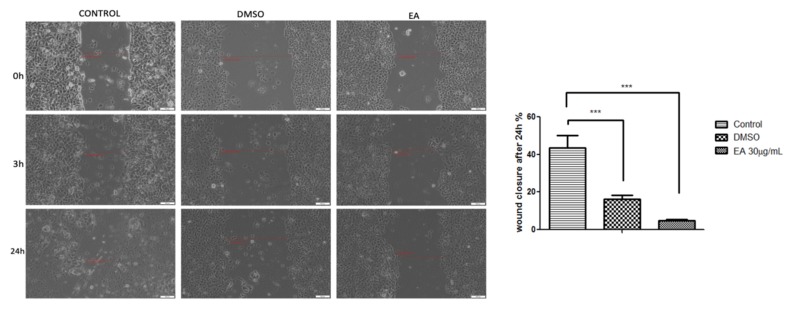
The migratory potential of MCF-7 human breast adenocarcinoma cells after treatment with EA extract at a concentration of 30 μg/mL. The pictures were taken by light microscopy, and the scale bars represent 100 μm. The bar graphs are expressed as percentage of wound healing after 24 h compared to the initial surface.

The potential anti-migratory activity of the EA on MCF-7 human breast adenocarcinoma cells was verified by means of a wound-healing technique, and the results are shown in Figure 4.

The results indicate that the EA extract had a strong inhibitory effect on the MCF-7 cells’ migration, showing a wound healing rate below 5% after an interval of 24 h. The solvent (DMSO) was also analysed for potential anti-migratory activity. It can be observed from Figure 4 that the solvent slowed down the migratory potential of the MCF-7 cells, inducing a wound closure percentage of almost 17%. The control cells (cells treated with growth medium) showed a closure of the scratch above 43%.

The cytotoxicity assessment revealed that the EA extract manifested a significant difference in the cytotoxic potential when compared to the positive control, displaying a cytotoxicity percentage above 13%. The solvent (DMSO) showed no significant cytotoxic potential on MCF-7 cells.

**Figure 5:**
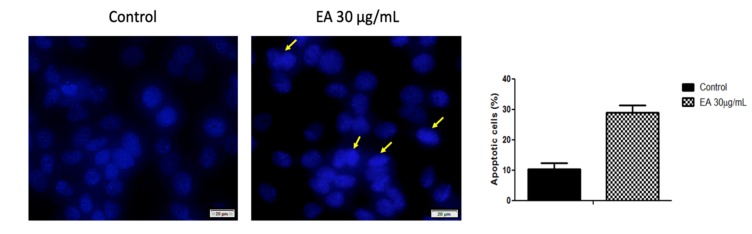
The effect of 30 μg/mL of EA on MCF-7 cells’ nuclei after 72 h. Morphological changes distinctive for apoptosis induction are marked with yellow arrows. For the nuclear visualization, DAPI staining was performed. The scale bars represent 20 μm.

In order to investigate the apoptotic potential of EA at the selected concentration, MCF-7 cells were treated with 30 μg/mL of EA for 72 h, and the cells’ nuclei were analyzed by DAPI staining. As shown in Figure 5, the control cells exhibit a normal organization, with a large, round nucleus, a clear nucleolus, and uniform chromatin density. However, after treatment, the MCF-7 cells manifested morphological changes distinctive for apoptosis induction, such as chromatin condensation.

**Figure 6:**
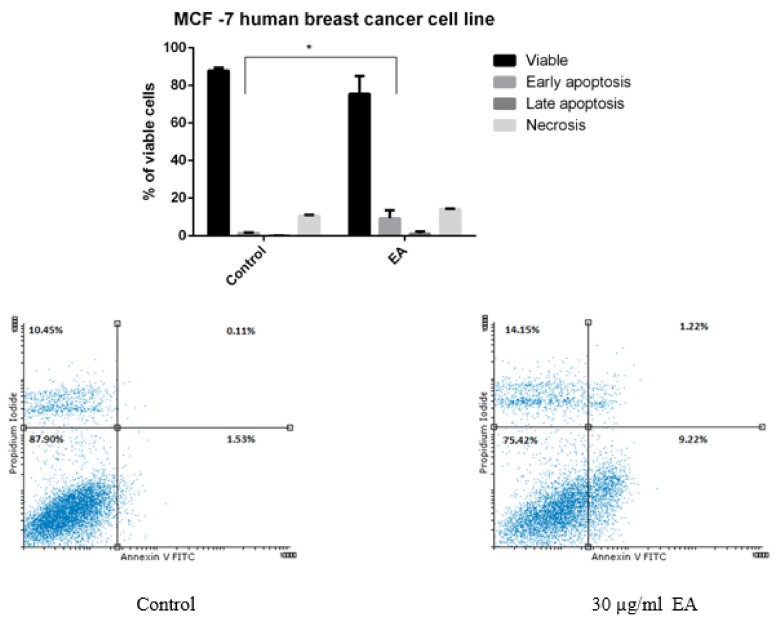
The evaluation of the pro-apoptotic activity of the MCF-7 human breast cancer cell line after 72 h of incubation with EA at the concentration of 30 µg/mL (lower right quadrant: early apoptotic cells; upper right quadrant: late apoptotic cells; upper left quadrant: necrotic cells).

Figure 6 presents the evaluation of the pro-apoptotic activity of the MCF-7 human breast cancer cell line after 72 h of incubation with EA at the concentration of 30 µg/mL (mean values) together with representative dot-plots. Early apoptosis, late apoptosis, and necrosis can be detected. The highest number of events was recorded for the process of early apoptosis.

## 3. Discussion

The results obtained in this study regarding the phytochemical composition—TA (17.57 mg/g extract), TP (156.22 mgGAE/g extract), and AA (7453 µmol Trolox/g)—are consistent with those reported by other authors.

In a comprehensive study on the phytochemical composition of different *Ephedra* species collected from the botanical garden of the University of Hamburg, Germany and analyzed as methanolic extracts, Ibragic and Sofic 2015 have reported the total phenolics content (TP, 53.3 ± 0.1 mg GAE/g dry weight) and total alkaloids content (TA, 0.2–15.24 mg E/g dry weight) [5].

The group of Jaradat et al. have analyzed the phytochemical composition of the same species collected from the mountains of Jenin region, Palestine using water, methanol, and ethanol for the extraction. The study reports that, when water was used, total polyphenols could not be detected in the extract, while small amounts of flavonoids were detected (0.519 ± 0.09 mg/g Rutin equivalent). Methanol was found to be the best solvent, leading to 47.62 ± 0.94 mg GAE/g dry weight of total polyphenols and 54.66 ± 0.12 mg/g Rutin equivalent of total flavonoids. Ethanol had a mild extraction capacity, leading to 19.175 ± 0.625 94 mg GAE/g dry weight of total polyphenols and 5.44 ± 0.625 mg/g Rutin equivalent of total flavonoids. The antioxidant activity of the extract was ascertained by a DPPH assay [17]. Jerbi et al. tested different solvents, namely hexane, ethyl acetat, and methanol, for the extraction, and recorded the highest percentage yield of extraction as well as the highest radical scavenging potential when methanol was employed [9]. It is well-known that evaporation leads to solvent removal; however, evaporation may not lead to 100% removal, as trace amounts of solvent can remain. Although the abovementioned studies showed that methanol extracts better than ethanol, which is an event that is expected due to polarity, we have chosen ethanol as the solvent in this study because of its lack of toxicity within an in vivo environment. In a similar approach, the group of Al-Rimawi et al. analyzed extracts of *Ephedra alata* Decne. collected from the southern part of the West Bank, Palestine using three different solvents, namely water, 100% ethanol, and 80% ethanol, in order to observe which solvent leads to the highest amount of total polyphenols (TP), the highest amount total flavonoids (TF), and the highest antioxidant activity (AA). The results showed that the polarity influences the screened parameters, with 80% ethanol being the best choice in terms of TP (101.2 ± 0.9 mg GAE/g dry weight) and AA (FRAP, 21.3 ± 0.4 mmol Fe+2/g dry weight; CUPRAC, 6442 ± 52 μmol Trolox/g dry weight; DPPH, 482.5 ± 1.7 μmol Trolox/g dry weight; and ABTS, 66.0 ± 1.5 μmol Trolox/g dry weight). On the other hand, the TF was higher in case of 100% ethanol (19.5 ± 0.3 mg catechin/g dry weight) [18].

The Ephedra genus is recognized for its high alkaloid content; in particular, alkaloids belonging to the ephedrine (E) type. However, a small number of studies point towards the composition of individual polyphenols. In this respect, this paper presents a new approach regarding the phytochemical composition of EA.

Amakura et al. reported on the main individual polyphenols in EA (cinnamic acid, syringin, catechin, epicatechin, symplocoside, pollenitin B, herbacetin 7-*O*-glucoside, kaempferol 3-*O*-rhamnoside 7-*O*-glucoside, and isovitexin 2-*O*-rhamnoside) [19]. The LC-DAD-ESI/MSn profile of *E. alata* was characterized by Ziani et al. It indicated the presence of 10 phenolic compounds, all belonging to the flavonoids class (five isoflavones and five flavones) [20]. Quercitin and kaempferol-3-*O*-rhamnoside have also previously been detected in *Ephedra* dietary supplements [21].

Our results on the polyphenols identified in the hydroalcoholic extracts of EA are consistent with previous data reported by other researchers, and indicate the presence of quercitin and kaempferol. The presence of rosmarinic acid in the hydroalcoholic extracts of EA represents a particularity that has not been reported in previous studies.

### Antimicrobial Activity

There is a paucity of studies in the literature on the antimicrobial activity of *Ephedra alata* Decne. A few groups, including that of Ghanem et al., have evaluated the antimicrobial activity of different types of extracts from *Ephedra alata*, namely water, methanol, and acetonitrile extracts, against four bacteria (*S. aureus*, *Pseudomonas aeruginosa*, *Bacillus subtilis*, and *Escherichia coli*) and four fungi (*Aspergillus fumigatus*, *Penicillium italicum*, *Syncephalastrum racemosum*, and *C. albicans*). The results showed that the only extract that was active against both Gram-positive and Gram-negative bacteria, as well as against fungi, was the acetonitrile extract. The methanolic extract elicited only antifungal potential, with *A. fumigatus* and *P. italicum* being the sensitive strains [22]. Good results for both Gram-positive and Gram-negative bacteria were obtained with the following solvents: butanol, ethyl acetate, and dichloromethane [23]. In this study, which analyses the ethanolic extract obtained from the aerial part of *Ephedra alata* Decne., we have shown that, among the tested strains, *S. aureus* and *Candida spp.* are the most sensitive strains to EA.

Some species of *Ephedra* have been assigned anticancer potential against various cell lines. For example, extracts obtained with different solvents from *Ephedra aphylla* have shown antiproliferative activity against the T47D and MCF-7 breast cancer cell lines. The same study showed that the extracts have weak antiproliferative potential against Vero, a normal kidney cell line [24]. *Ephedra campylopoda* stem methanolic and ethanolic extracts elicited an antiproliferative effect on the Jurkat human leukemic T cell line [25]. The herbal extract of *Ephedra* initially obtained from *Ephedra sinica*, as well as an ephedrine alkaloid-free *Ephedra* herb extract, has shown antiproliferative potential against the H1975 non-small-cell lung cancer cell line [26]. Mohamad et al. have presented data on the antiproliferative effect of *Ephedra campylopoda* collected from the south of Lebanon on HT-29 human epithelial cells and HCT116 human colon cancer cells [14]. The cytotoxic effect (at 24 h of incubation) and cytostatic effect (at 72 h of incubation) of *Ephedra alata* extracts in the range of 0–1000 μg/mL were analyzed for monocultures and co-cultures of Hepg2 and THP-1-derived macrophages. Significant cytotoxic effects were recorded for concentrations higher than 500 μg/mL. The cytostatic activity was stronger in the co-cultures (an IC_50_ of 380 μg/mL) [27]. Mendelovich et al. have shown that ethanolic extracts from the leaves, as well as the fruit juice, of *Ephedra foeminea* have significant antiproliferative and pro-apoptotic potential against MDA-MB-231 (human breast cancer), HCT116 (human colon) and HaCaT (human keratinocytes) cells [28]. In an ethnopharmacological study, Jaradat et al. concluded, based on questionnaires addressed to women diagnosed with breast cancer on the West Bank of Palestine, that the leaves and seeds of *Ephedra alata*, mostly prepared in the form of a decoction, are the most frequently used vegetal products in the treatment of breast cancer [29]. In a more comprehensive ethnopharmacological study conducted in the same region that included 150 herbalists, traditional healers, and rural dwellers, Jaradat et al. statistically observed that 72 plants have been employed for treatment of this pathology, with the most represented families being *Compositae* and *Lamiaceae*. The analysis showed that lung cancer was the type of malignancy most often treated with plants, and that *Ephedra alata* was the most frequently used phytomedicine for the management of cancer in Palestine [30,31]. The novelty of this study from the point of view of anticancer evaluation is the fact that, in the set experimental parameters, EA exhibits important antiproliferative, and weak pro-apoptotic and cytotoxic, potential against the MCF-7 human breast cancer cell line. So, the results from the abovementioned ethnopharmacological studies are supported by our experimental data, which attest to the in vitro anticancer activity of EA by mechanisms that involve the inhibition of proliferation.

## 4. Materials and Methods

### 4.1. Plant Materials

Dried aerial parts of *Ephedra alata* Decne. were bought in Romania from the southern part of Tunisia (Djerba) by a student of the Victor Babeş University of Medicine and Pharmacy, identified in the department of Pharmacognosy, and assigned the voucher specimen code Ea 12/2018. Some of the dried aerial parts were ground with the help of a commercial blender. From the powder, 10 g were weighted, and 50 mL of 70% ethanol were added. The extraction was conducted in an ultrasonic bath (FALC LBS2, Treviglio, Italia) for 30 min at a frequency of 40 kHz and a temperature of 50 ° C. The extract was then filtered with a vacuum pump. The solvent was evaporated to dryness in a rotary evaporator (250 bar pressure, temperature 60 ° C, and 150 rpm). The extract (EA) was stored at −4 ° C until use.

### 4.2. Determination of Total Polyphenols Content (TP)

From the previously obtained extract, 0.1 g were dissolved in 1 mL methanol and ultrasonicated for 30 min using an Aqua Wave 9381 (Barnstead Lab Line, Thermo Fisher Inc.). A 0.5 mL amount of alcoholic extract was treated with 1.25 mL of Folin–Ciocalteu (Merck, Darmstadt, Germany) reagent diluted 1:10 with water. The sample was incubated for 5 mins at room temperature, and 1 mL of 60 g/L Na_2_CO_3_ (S.C.Chemical Company S.A., Bucuresti, Romania) was added. After 30 min of incubation at 50 °C, the absorbance of the samples was measured at 750 nm using a UV-VIS spectrophotometer (Analytic Jena Specord 205, Jena, Germany). The calibration curve was obtained using gallic acid (GA) (Sigma Aldrich Chemie, Madrid, Spain) as a standard (concentration range 5–250 μg/mL) and a blank ethanol control (Sigma-Aldrich; Merck KGaA, Darmstadt, Germany). The regression equation was: y = 1.92x − 0.10, and the coefficient of correlation R^2^ = 0.9980. The results were expressed in mg GAE·g^−1^ extract. All experiments were performed in triplicate.

### 4.3. Determination of Individual Polyphenols by LC-MS

For individual polyphenols identification, two hydroalcoholic extracts of EA were tested. The first extract was obtained in a concentration of 50 mg/mL after dissolving the extract prepared according to Section 4.1. in 70% ethanol. The second extract was obtained in a concentration of 10 mg/mL in methanol:water:formic acid (90:8:2, *v/v/v*). For the determination of individual polyphenols, a Shimadzu Chromatograph equipped with SPD-10A UV and LC-MS 2010 detectors was used. The chromatographic conditions were as follows: mobile phases A: water acidified with formic acid at pH 3; B: acetonitrile acidified with formic acid at pH 3; gradient program: 0.01–20 min, 5% B; 20.01–50 min, 5–40% B; 50–55 min, 40–95% B; and 55–60 min 95% B. The solvent flow rate was 0.3 ml/min at 20 °C. The monitoring wavelength was 280–320 nm. Two chromatographic columns were used: an Adsorbosphere UHS C18, 5 µm, Lot no.007250 (Column I) and an EC 150/2 NUCLEODUR C18 Gravity SB 150 × 2 mm × 5 μm column, ref:760618.20, SN E 15110907, Lot 38775055 (Column II). The calibration curves were performed in the range of 20–50 μg/mL. The results were expressed in mg∙g^−1^ d.m. Experiments were performed in duplicate. All standards were prepared in methanol (Merck KGaA), and all reagents and solvents used were of analytical grade. Standards of polyphenols were purchased from Sigma-Aldrich, Merck KGaA. The calibration curve and *m/z* signal are presented in Table 1.

### 4.4. Determination of the Total Antioxidant Activity (AA)

For the determination of the antioxidant activity (AA), the CUPRAC method was employed. The CUPRAC method is a spectrophotometric technique that depends upon the reduction of a cupric neocuproine complex to a cuprous neocuproine complex by a reductant at low pH [32]. The neocuproine complex can be monitored at 450 nm. As a reference substance, Trolox (6-hydroxy-2,5,7,8-tetramethilcroman-2-carboxylic acid) (Sigma-Aldrich; Merck KGaA, Darmstadt, Germany), an antioxidant with a structure similar to vitamin E, was used. Reagents: 0.01 M CuCl2 (S.C.Chemical Company S.A., Bucuresti, Romania), 7.5 × 10^−3^ M, neocuproine (2,9-Dimethyl-1,10-phenanthroline) (Sigma–Aldrich, Germany), and acetate buffer. One milliliter (1 mL) of 0.01 M CuCl2 solution was mixed with 1 mL neocuproine (7.5 × 10^−3^) and 1 mL of acetate buffer. At this solution, 1.1 mL of sample (alcoholic extract) was added. For the blank, 50% ethanol was used. The absorption was read after 0.5 hours at 20 °C at 450 nm. All determinations were performed in triplicate.

### 4.5. Determination of Total Alkaloids (TA)

The determination of total alkaloids (TA) was done using the ninhydrin reaction according the method described by Ibragic and Sofic 2015 [5]. Ninhydrin reacts with aminoacid groups and yields a violet-blue compound with a maximum absorbance at 570 nm. Ninhydrin solution (2%) was prepared in ethanol and adjusted to pH 7.8–7.9 with Na_2_CO_3_ 6 g/L. One hundred microliters (100 µL) of ninhydrin solution was added to 100 µL of extract, obtained as described in Section 4.1., and the volume was filled to 1 mL with ethanol. The absorbance of the coloured compound was read at 570 nm using a UV-VIS spectrophotometer (Analytic Jena Specord 205). The calibration curve was obtained from Ephedrine stock solution (50 mg/mL, Zentiva SA, Bucuresti, Romania). It was used to obtain serial standard dilution samples in a concentration that ranged between 12 and 32 µg/mL. The determinations were performed in triplicate. The coefficient of correlation for the calibration curve was R^2^ = 0.9547. The results were expressed in mg ephedrine/g extract.

### 4.6. In Vitro Antimicrobial Activity

The EA at the concentration of 30 µg/mL was screened for its antimicrobial activity against seven bacterial and two fungus strains. Reference strains see Table 5.

### 4.7. Disk Diffusion Method

The antimicrobial activity of the extract was evaluated, as previously described, by the agar disk diffusion method [33,34]. Mueller–Hinton agar plates (Sanimed, Bucharest, Romania) were inoculated with 100 µL of a microbial suspension (10^8^ germs/mL) in physiological saline. Ten microliters, from each sample, were placed on a blank paper disk (BioMaxima, Lublin, Poland), then deposited onto the surface of the cultured media. Agar plates inoculated with the microbial suspensions were incubated at 37 °C for 24 h. The reading of the inhibition diameters was made in millimeters with a ruler. For all bacterial strains, the disk-diffusion tests were performed in triplicate. The positive control was represented by gentamycin or fluconazole disks (Bio-Rad, Marnes-la-Coquette, France). For the negative control, we used a blank paper disk impregnated with DMSO.

### 4.8. Determination of the Minimum Inhibitory Concentration (MIC) and the Minimum Bactericidal Concentration (MBC)

The EA was tested by a broth dilution assay, as recommended by the Clinical Laboratory and Standard Institute (CLSI). A concentration of 200 μg/Ml was prepared. In five test tubes, serial 2-fold dilutions in Mueller–Hinton broth (Sanimed, Bucharest, Romania) of the EA were done. The test tubes were inoculated with 5 × 10^5^ bacteria/ml. After incubating the test tubes at 37 °C for 24 h, the MIC (the lowest concentration that yielded no growth) was determined. In addition, the test tubes with no visible growth were inoculated on Columbia agar +5% sheep’s blood or Sabouraud chloramphenicol agar (Sanimed, Bucharest, Romania) in order to determine the MBC (the lowest concentration that killed 99.9% of the initial inoculum). The medium plates were incubated at 37 °C for 24 h, and the MBC was determined [35].

### 4.9. Cell Culture

The cells used in this study were MCF-7 human breast cancer cells purchased from ATCC (American Type Culture Collection; code no ATCC^®^ HTB-22^TM^) as a frozen vial. The cell line was cultured in specific growth medium Eagle’s Minimum Essential Medium (EMEM, code no ATCC^®^ 30-2003^™^) supplemented with 10% FBS (fetal bovine serum) and 1% antibiotics mixture of penicillin/streptomycin (Sigma-Aldrich, Munich, Germany). The cells were maintained in a standard condition: a humidified atmosphere with 5% CO_2_ at 37 °C in a Steri-Cycle i160 incubator (Thermo Fisher Scientific, USA), and supervised daily.

### 4.10. Antiproliferative MTT Assay

The assessment of the antiproliferative activity was conducted as previously described [36]. The cells were plated at a density of 5000 cells/well using 96-well plates and incubated with selected concentrations of EA. After 72 h of incubation, 5 mg/mL MTT (3-(4,5-dimethylthiazol-2-yl)-2,5-diphenyltetrazolium bromide) solution was added and incubated for another 4 h. The absorbance of the precipitated formazan crystals dissolved in dimethyl sulfoxide (DMSO) was measured with a microplate reader at 545 nm. Wells with cells incubated with medium and DMSO were used as a control [37]. The results are presented as the mean of three different experiments.

### 4.11. Anti-Migratory Potential: A Wound Healing Technique

In order to study the migration of MCF-7 cells after treatment with the EA extract and DMSO, respectively, the in vitro scratch assay method was applied. This is an economical, accessible, and undemanding technique to express cell-to-cell interactions [38,39].

A number of 2 × 10^5^ cells was plated onto a 12-well culture plate and allowed to attach to the bottom of the plate until a confluent cell monolayer was formed. After that, an artificial gap was made with a sterile tip in the middle of each well. All the cellular debris and detached cells were washed with 1.5 mL PBS/well. Further, the cells were treated with EA extract (30 μg/mL) and DMSO, used in an equal volume, as the test compound. The gap filling was supervised by taking pictures of the scratched area at 0, 3, and 24 h. Photographs were performed at a magnification of 10× using an inverted microscope (Olympus IX73, Tokyo, Japan) equipped with a DP74 camera. Scratch surfaces were analyzed with the CellSense Dimension software (Version 1.17, Olympus, Tokyo, Japan) and the migration percentage was determined according to the formula described by Felice et al. [40].

### 4.12. Determination of the Cytotoxic Potential by the Means of Lactate Dehydrogenase (LDH) Assay

The cytotoxic rate of the EA extract at the highest concentration (30 μg/mL) was determined by quantification of the LDH leakage into the media when cellular membrane damage emerged. The protocol was almost the same as the one used for the cell viability assessment, with the exception that, on the day of the assay, 50 μL from each well was transferred to a new 96-well plate and mixed with reaction mixture (50 μL/well), followed by an incubation of 30 min at room temperature. After this step, 50 μL/well of stop solution was added, and the level of LDH released into the medium was determined by measuring the absorbance of the wells at the wavelengths of 490 nm and 680 nm using a microplate reader (xMark^TM^ Microplate, Biorad). A Positive Control was prepared by combining 1 μL of LDH Positive Control with 10 mL of FCS in PBS (to a final concentration of 1% FCS).

### 4.13. DAPI: Cell Nuclei Staining

MCF-7 cells cultured in a 6-well plate were stimulated with EA extract (30 μg/mL) for 72 h. At the end of the incubation period, the cells were washed two times with ice-cold PBS and fixed with 4% paraformaldehyde in PBS for 30 min. Fixed cells were washed again with PBS, permeabilized with 2% Triton X-100 (Sigma) for 30 min, and blocked with 30% FCS in 0.01% Triton X-100 for 1 h. The final step consisted in staining the cells with DAPI (4’,6’-diamidino–2-phenylindole) in a dark chamber for 15 min. Cell nuclei were analyzed at a magnification of 40× with a fluorescence Olympus IX73 microscope equipped with an integrated DP74 camera (Olympus, Tokyo, Japan).

### 4.14. Annexin V-PI Assay

A number of 5 × 10^5^ cells/well were seeded into a 6-well plate (Greiner bio-one) and left overnight in order to attach to the bottom of the plate. After 24 h, the cultured medium was removed, and a fresh medium containing the tested extracts at a concentration of 30 µg/mL was added. The final concentration of the tested compounds was obtained by successive dilutions into the culture medium, starting from a stock solution of 10 mg/mL (extract) in DMSO. Untreated cells were used as a control; cells treated with DMSO were used as a solvent control. After 72 h, the cells were trypsinized and analyzed for the apoptotic effect of EA extract using flow cytometry. Annexin V-FITC combined with a propidium iodide (PI) kit (Invitrogen, ThermoFisher, Vienna, Austria) was used in the cell death flow cytometric studies (apoptosis) following the manufacturer’s protocol. Briefly, 2–5 × 10^5^ cells were washed two times in 1× Annexin V Binding Buffer, centrifuged at 1500 RPM for 5 min, resuspended in the binding buffer, and incubated with 5 μL of Annexin V-FITC for 15 min in the dark. After washing the cells with 200 μL specific binding buffer and centrifugation, the cell pellet was resuspended in 190 μL binding buffer, and 10 μL of PI solution was added immediately prior to the analysis by flow cytometry. The results are presented as the mean of three different experiments ± standard deviation.

### 4.15. Statistics

The Prism software package GraphPad Prism 5.01 (GraphPad Software, San Diego, CA, USA) was used for data collection and presentation. The data ranged from three to six separate experiments, and are presented as mean ± SD. Statistical significance was assessed by one-way ANOVA fallowed by a Newman–Keuls post-hoc test for the comparison of multiple groups. *, **, ***, and **** indicate *p* < 0.05, *p* < 0.01, *p* < 0.001, and *p* < 0.0001, respectively, compared to the control group.

## 5. Conclusions

Ethanolic extracts of the aerial part of *Ephedra alata* Decne., which is indigenous to the southern part of Tunisia (Djerba), contain polyphenolic phytocompounds and elicit antioxidant activity. The LC-MS results show that, among the screened polyphenolic compounds, the extract contains kaempherol, epicatechin, and quercetin. The extract acts as a bacteriostatic agent against all tested bacterial strains (*K*. *pneumoniae*, → *S*. *flexneri, S.enterica*, → *E. coli*, *P.aeruginosa*, → *S*. *aureus*, *E*. *faecalis C*. *albicans,* and *.parapsilosis*); however, it is bactericidal only against the Gram-positive cocci and *Candida* spp. In the set of experimental conditions, the hydroalcoholic extract has a potential antiproliferative, pro-apoptotic, and cytotoxic effect against the MCF-7 human breast cancer cell line.

## Figures and Tables

**Table 1:** The total phenolic content (TP), antioxidant activity (AA), and total alkaloids (TA) of the hydroalcoholic extract of the aerial part of *Ephedra alata* Decne. (EA).

Sample	TP (mgGAE/g Extract)	AA (μmol Trolox/g Extract)	TA (mg Ephedrine/g Extract)
EA	156.226 ± 0.5	7453.18 ± 2.5	17.57 ± 0.6

**Table 2:** The individual polyphenols of the hydroalcoholic extract of the aerial part of *Ephedra alata* Decne. (EA), and the LC-MS parameters.

Compounds	Retention Time Column I (min)	Retention Time Column II (min)	m/z Signal	EA in Hydroethanolic Extract		EA in Hydromethanolic Extract Chromatographic Column I	Calibration Curve
				Column I	Column II		
				µg/mL ± SD (µg/mg)	µg/mL ± SD (µg/mg)	(µg/mL ± SD) (µg/mg)	
Gallic acid	4.826	4.745	169	nd	nd	nd	y = 8.470 e-006x (r = 0.9996);
Protocatecuic acid	11.982	11.774	153	nd	nd	nd	y = 8.036 e-006x (r = 0.9990);
Caffeic acid	18.605	18.396	179	0.709 ± 0.279 (0.014)	nd	0.389 ± 0.49 (0.008)	y = 7.110 e-006x (r = 0.9990);
Epicatechin	22.910	23.242	289	nd	7.736 ± 0.297 (0.155)	nd	y = 3.881 e-005x (r = 0.9996);
p-coumaric acid	24.374	24.454	163	0.241 ± 0.028 (0.005)	nd	0.261 ± 0.021 (0.005)	y = 1.1566 e-006x (r = 0.9997);
Ferulic acid	26.333	24.883	193	nd	nd	nd	y = 1.172 e-006x (r = 0.9999);
Rutin	27.290	26.086	609	nd	nd	nd	y = 1.813 e-005x (r = 0.9999)
Rosmarinic acid	28.252	29.129	359	0.915 ± 0.008 (0.018)	0.397 ± 0.002 (0.008)	0.807 ± 0.049 (0.016)	y = 1.018 e-006x (r = 0.9982);
Resveratrol	29.749	29.887	227	9.403 ± 0.008 (0.188)	12.963 ± 0.454 (0.259)	10.387 ± 0.422 (0.207)	y = 6.388 e-006x (r = 0.9945);
Quercitin	32.591	31.841	301	2.873 ± 0.70 (0.057)	2.387 ± 0.06 (0.048)	4.572 ± 0.384 (0.091)	y = 1.001 e-005x (r = 0.9992);
Kaempferol	34.470	34.378	285	24.297 ± 2.73 (0.485)	6.814 ± 0.179 (0.136)	28.675 ± 2.579 (2.867)	y = 3.273 e-005x (r = 0.9990).

nd = not detected.

**Table 3:** The inhibition diameters for selected strains after incubation with 30 µg/ml of EA.

Compound	*Klebsiella Pneumonia*	*Shigella flexneri*	*Salmonella Enterica*	*Escherichia coli*	*Pseudomonas Aeruginosa*	*Staphylococcus Aureus*	*Enterococcus Faecalis*	*Candida Albicans*	*Candida Parapsilosis*
*EA*	7 mm	7 mm	7 mm	7 mm	7 mm	9 mm	7 mm	10 mm	10 mm

**Table 4:** The Results on the minimum inhibitory concentration (MIC) and the minimum bactericidal concentration (MBC).

Species	MIC (μg/mL)	MBC (μg/mL)
*K. pneumoniae*	200	-
*S. flexne*	200	-
*S. enterica*	200	-
*E. coli*	200	-
*P. aeruginosa*	200	-
*S. aureus*	50	100
*E. faecalis*	100	200
*C. albicans*	50	100
*C. parapsilosis*	50	100

**Table 5:** Reference strains.

Bacterial Species	ATCC	Producer
*Salmonella enterica* serotype *typhimurium*	14,028	Thermo Scientific
*Shigella flexneri* serotype 2b	12,022	Thermo Scientific
*Enterococcus faecalis*	51,299	Thermo Scientific
*Escherichia coli*	25,922	Thermo Scientific
*Klebsiella pneumoniae*	700,603	Thermo Scientific
*Pseudomonas aeruginosa*	27,853	Thermo Scientific
*Staphylococcus aureus*	25,923	Thermo Scientific
*Candida albicans*	10,231	Thermo Scientific
*Candida parapsilosis*	22,019	Thermo Scientific

## References

[1] Atanasov A.G., Waltenberger B., Pferschy-Wenzig E.-M., Linder T., Wawrosch C., Uhrin P., Temml V., Wang L., Schwaiger S., Heiss E.H. (2015). Discovery and resupply of pharmacologically active plant-derived natural products: A review. Biotechnol. Adv..

[2] Rydin C., Pedersen K.R., Crane P.R., Friis E.M. (2006). Former diversity of Ephedra (Gnetales): Evidence from Early Cretaceous seeds from Portugal and North America. Ann. Bot..

[3] Hegazi G.A.E.M., El-Lamey T.M. (2011). In vitro production of some phenolic compounds from Ephedra alata Decne. J. Appl. Environ. Biol. Sci..

[4] Caveney S., Charlet D.A., Freitag H., Maier-Stolte M., Starratt A.N. (2001). New observations on the secondary chemistry of world Ephedra (Ephedraceae). Am. J. Bot..

[5] Ibragic S., Sofić E. (2015). Chemical composition of various Ephedra species. Bosn. J. Basic Med. Sci..

[6] Abourashed E.A., El-Alfy A.T., Khan I.A., Walker L. (2003). Ephedra in perspective—A current review. Phytother. Res..

[7] Magalhães E., Govêia C.S., Ladeira L.C.d.A., Nascimento B.G., Kluthcouski S.M.C. (2009). Ephedrine versus phenylephrine: Prevention of hypotension during spinal block for cesarean section and effects on the fetus. Braz. J. Anesthesiol..

[8] Cocan I., Alexa E., Danciu C., Radulov I., Galuscan A., Obistioiu D., Morvay A.A., Sumalan R.M., Poiana M.A., Pop G. (2018). Phytochemical screening and biological activity of *Lamiaceae* family plant extracts. Exp. Ther. Med..

[9] Jerbi A., Zehri S., Abdnnabi R., Gharsallah N., Kammoun M. (2016). Essential oil composition, free-radical-scavenging and antibacterial effect from stems of Ephedra alata alenda in Tunisia. J. Essent. Oil Bear. Plants.

[10] Parsaeimehr A., Sargsyan E., Javidnia K. (2010). A comparative study of the antibacterial, antifungal and antioxidant activity and total content of phenolic compounds of cell cultures and wild plants of three endemic species of Ephedra. Molecules.

[11] Khan A., Jan G., Khan A., Gul Jan F., Bahadur A., Danish M. (2017). *In vitro* antioxidant and antimicrobial activities of Ephedra gerardiana (root and stem) crude extract and fractions. Evid.-Based Complement. Altern. Med..

[12] Pullela S.V., Takamatsu S., Khan S.I., Khan IA. (2005). Isolation of lignans and biological activity studies of Ephedra viridis. Planta Med..

[13] Nam N.-H., Lee C.-W., Hong D.-H., Kim H.-M., Bae K.-H., Ahn B.-Z. (2003). Antiinvasive, antiangiogenic and antitumour activity of Ephedra sinica extract. Phytother. Res..

[14] Mohamad N., Falah A., Fatima J., Hussein K., Akram H., Ali C., Hassan R. (2016). Antibacterial, antioxidant and antiproliferative activities of the hydroalcoholic extract of the lebanese plant: Ephedra Campylopoda. Int. Res. J. Pharm..

[15] Attard E., Vella K. (2009). Effects of ephedrine and Ephedra fragilis crude extracts on human peripheral lymphocytes. Pharmacogn. Res..

[16] Al-Qarawi A.A., Abd-Allah E.F., Hashem A. (2011). Ephedra alata as biologically-based strategy inhibit aflatoxigenic seedborne mold. Afr. J. Microbiol. Res..

[17] Jaradat N., Hussen F., Anas A.A. (2015). Preliminary phytochemical screening, quantitative estimation of total flavonoids, total phenols and antioxidant activity of Ephedra alata Decne. J. Mater. Environ. Sci..

[18] Al-Rimawi F., Abu-Lafi S., Abbadi J., Alamarneh A.A.A., Sawahreh R.A., Odeh I. (2017). Analysis of phenolic and flavonoids of wild Ephedra alata plant extracts by LC/PDA and LC/MS and their antioxidant activity. Afr. J. Tradit. Complement. Altern. Med..

[19] Amakura Y., Yoshimura M., Yamakami S., Yoshida T., Wakana D., Hyuga M., Hyuga S., Hanawa T., Goda Y. (2013). Characterization of Phenolic Constituents from Ephedra Herb Extract. Molecules.

[20] Ziani B.E.C., Heleno S.A., Bachari K., Dias M.I., Ferreira I.C.F. (2018). Phenolic compounds characterization by LC-DAD-ESI/MSn and bioactive properties of Thymus algeriensis Boiss. & Reut. and Ephedra alata Decne. Food Res. Int..

[21] Grippo A.A., Capps K., Rougeau B., Gurley B.J. (2007). Analysis of flavonoid phytoestrogens in botanical and ephedra-containing dietary supplements. Ann. Pharmacother..

[22] Ghanem S., El-Magly U.I.A. (2008). Antimicrobial activity and tentative identification of active compounds from the medicinal Ephedra alata male plant. J. Taibah Univ. Med. Sci..

[23] Chebouat E., Dadamoussa B., Gharabli S., Gherraf N., Allaoui M., Cheriti A., Lahham A., Zellagui A. (2014). Assessment of antimicrobial activity of flavonoids extract from Ephedra alata. Der Pharmacia Lettre.

[24] Al-Awaida W., Al-Hourani B.J., Akash M., Talib W.H., Zein S., Falah R.R. (2018). In vitro anticancer, anti-inflammatory, and antioxidant potentials of Ephedra aphylla. J. Cancer Res. Ther..

[25] Kallassy H., Fayyad-Kazan M., Makki R., EL-Makhour Y., Rammal H., Leger D.Y., Sol V., Fayyad-Kazan H., Liagre B., Badran B. (2017). Chemical composition and antioxidant, anti-inflammatory, and antiproliferative activities of Lebanese Ephedra Campylopoda plant. Med. Sci. Monit. Basic Res..

[26] Oshima N., Yamashita T., Hyuga S., Hyuga M., Kamakura H., Yoshimura M., Maruyama T., Hakamatsuka T., Amakura Y., Hanawa T. (2016). Efficiently prepared ephedrine alkaloids-free Ephedra Herb extract: A putative marker and antiproliferative effects. J. Nat. Med..

[27] Kmail A., Lyoussi B., Zaid H., Saad B. (2017). In vitro assessments of cytotoxic and cytostatic effects of Asparagus aphyllus, Crataegus aronia, and Ephedra alata in monocultures and co-cultures of Hepg2 and THP-1-derived macrophages. Pharmacogn. Commun..

[28] Mendelovich M., Shoshan M., Fridlender M., Mazuz M., Namder D., Nallathambi R., Selvaraj G., Kumari P., Ion A., Wininger S. (2017). Effect of Ephedra foeminea active compounds on cell viability and actin structures in cancer cell lines. J. Med. Plant Res..

[29] Jaradat N.A., Shawahna R., Eid A.M., Al-Ramahi R., Asma M.K., Zaid AN. (2016). Herbal remedies use by breast cancer patients in the West Bank of Palestine. J. Ethnopharmacol..

[30] Jaradat N.A., Al-Ramahi R., Zaid A.N., Ayesh O.I., Eid A.M. (2016). Ethnopharmacological survey of herbal remedies used for treatment of various types of cancer and their methods of preparations in the West Bank-Palestine. BMC Complement. Altern. Med..

[31] Abu-Darwish M.S., Efferth T. (2018). Medicinal plants from Near East for cancer therapy. Front. Pharmacol..

[32] Atak M., Mavi K., Uremis I. (2016). Bio-herbicidal effects of oregano and rosemary essential oils on germination and seedling growth of bread wheat cultivars and weeds. Rom. Biotechnol. Lett..

[33] Pavel I.Z., Danciu C., Oprean C., Dehelean C.A., Muntean D., Csuk R., Muntean D.M. (2016). In vitro evaluation of the antimicrobial ability and cytotoxicity on two melanoma cell lines of a benzylamide derivative of maslinic acid. Anal. Cell. Pathol. (Amst.).

[34] Oprean C., Zambori C., Borcan F., Soica C., Zupko I., Minorics R., Bojin F., Ambrus R., Muntean D., Danciu C. (2016). Anti-proliferative and antibacterial in vitro evaluation of the polyurethane nanostructures incorporating pentacyclic triterpeneS. Pharm. Biol..

[35] Wikler M.A., Low D.E., Cockerill F.R., Sheehan D.J., Craig W.A., Tenover F.C. (2006). Methods for Dilution Antimicrobial Susceptibility Tests for Bacteria That Grow Aerobically; Approved Standard.

[36] Szabó J., Jerkovics N., Schneider G., Wölfling J., Bózsity N., Minorics R., Zupkó I., Mernyák E. (2016). Synthesis and in vitro antiproliferative evaluation ofC-13 epimers of triazolyl-D-secoestrone alcohols: The first potent 13a-D-secoestrone derivative. Molecules.

[37] Mosmann T. (1983). Rapid colorimetric assay for cellular growth and survival: Application to proliferation and cytotoxicity assays. J. Immunol. Methods.

[38] Liang C.C., Park A.Y., Guan J.L. (2007). In vitro scratch assay: A convenient and inexpensive method for analysis of cell migration in vitro. Nat. Protoc..

[39] Moacă E.A., Farcaş C., Ghiţu A., Coricovac D., Popovici R., Cărăba-Meiţă N.L., Ardelean F., Antal D.S., Dehelean C., Avram Ş. (2018). A Comparative Study of Melissa officinalis Leaves and Stems Ethanolic Extracts in terms of Antioxidant, Cytotoxic, and Antiproliferative Potential. Evid. Based Complement. Alternat. Med..

[40] Felice F., Zambito Y., Belardinelli E., Fabiano A., Santoni T., Di Stefano R. (2015). Effect of different chitosan derivatives on in vitro scratch wound assay: A comparative study. Int. J. Biol. Macromol..

